# Changes in the Expression of Inflammatory Genes Induced by Chronic Exercise in the Adipose Tissue: Differences by Sex

**DOI:** 10.3390/sports12070184

**Published:** 2024-07-01

**Authors:** Paula Sanchis, Aida Ezequiel-Rodriguez, Antonio Jesús Sánchez-Oliver, Walter Suarez-Carmona, Sergio Lopez-Martín, Francisco José García-Muriana, José Antonio González-Jurado

**Affiliations:** 1 Centre for Physical Activity Research, 2100 Copenhagen, Denmark; 2 Faculty of Sport Science, Universidad Pablo de Olavide, 41013 Seville, Spain; 3 Departamento de Motricidad Humana y Rendimiento Deportivo, University of Seville, 41013 Seville, Spain; 4 Department of Cell Biology, Faculty of Biology, University of Seville, 41012 Seville, Spain; 5 Laboratory of Cellular and Molecular Nutrition, Instituto de la Grasa, CSIC, 41013 Seville, Spain; 6 Research Center on Physical and Sports Performance, Universidad Pablo de Olavide, 41013 Seville, Spain

**Keywords:** obesity, concurrent training program, inflammatory markers, gene expression, gene regulation

## Abstract

The impact of obesity on adipose tissue function is well acknowledged, but the role of physical exercise in regulating inflammatory markers and gene expression in obese individuals remains uncertain. This study aims to investigate the effects of chronic exercise on inflammatory gene expression in adipose tissue and to explore sex differences in response to exercise. The study involved 29 obese participants (13 men, 16 women) aged 38 to 54 years with a mean BMI of 36.05 ± 4.99 kg/m^2^. Participants underwent an 8-week concurrent training program comprising three weekly sessions of ~60 min each. The sessions included joint mobility exercises, cardiovascular activation, and cardiorespiratory resistance exercises at medium to low intensity. A fine-needle aspiration biopsy of abdominal subcutaneous adipose tissue was performed for gene expression analysis using quantitative polymerase chain reaction (qPCR). The study demonstrated that chronic exercise modulates the expression of pro-inflammatory genes in subcutaneous adipose tissue, particularly ADIPOR2 (*p* = 0.028), leptin (*p* = 0.041), and IFNg (*p* = 0.040) (downregulated). Interestingly, regardless of sex, the exercise programs had an independent effect on pro-inflammatory genes. Overall, this study provides insight into the role of chronic exercise in modulating adipose tissue gene expression in obese individuals. Further research involving both sexes is recommended to tailor exercise interventions for better outcomes.

## 1. Introduction

Obesity is a pandemic problem that results from excessive food intake combined with low levels of physical activity, leading to excessive accumulation of adipose tissue and subsequent weight gain [1]. The WHO European Regional Obesity Report 2022 shows that 59% of adults and 30% of children in Europe are overweight or living with obesity [2]. Given that obesity could be controlled by lifestyle changes, such as regular physical exercise, it is crucial to understand how physical activity can regulate the underlying mechanism of improved metabolic health [3].

There has been a growing interest in the regulation of gene expression by exercise due to its biochemical and metabolic benefits [4]. In this sense, preclinical studies have shown that a single bout of exercise induces changes in the expression of muscle genes related to mitochondrial biogenesis [5], oxidative phosphorylation (OXPHOS) [6], antioxidant mechanisms [7], and insulin resistance [8], as well as changes in white adipose tissue (WAT) genes involved in browning [9]. In addition, there is considerable evidence to suggest that metabolic health benefits may be the result of training adaptations that occur in response to repeated exposure to exercise-induced transient transcriptional responses after each training session [10]. Therefore, to gain new insights into the underlying mechanisms of chronic adaptations, it is essential to determine how exercise induces the favourable remodelling of different tissues to reduce the risk of developing obesity and metabolic diseases. For instance, Chapman et al. showed that long-term exercise training in humans could shift the gene expression of several genes in muscles to attenuate metabolic diseases [11]. Concerning adipose tissue, and in particular WAT, the scientific community is only beginning to explore the molecular mechanisms underlying long-term physical activity [12].

The traditional role of WAT has been as an energy storer, releasing fatty acids when fuel is required. In particular, subcutaneous WAT has been reported to be primarily responsible for fatty acids released into the systemic circulation at rest and during exercise. However, visceral WAT contributes much less to the systemic fatty acid pool [13,14]. Emerging research suggests that WAT is a major secretory and endocrine organ with distinct functions beyond simple fat storage [15]. In fact, WAT is the main source of low-grade inflammatory metabolism in obese individuals [16] and is associated with a chronic low-grade inflammatory metabolic state that leads to the overall increased development of a huge range of chronic diseases such as type 2 diabetes mellitus, dyslipidaemia, and cardiovascular disease [17,18,19]. Given the WAT responses to changes in physical activity, elucidating whether exercise can attenuate systemic WAT inflammation independent of fat loss is crucial to understanding how chronic physical activity might affect health outcomes. Using preclinical models, it has long been recognised that physical activity reduces adipocyte size and modulates the gene expression of pro-inflammatory adipokines, thereby attenuating low-grade inflammation and improving insulin sensitivity [20,21]. In humans, exercise interventions have remarkable benefits on fat metabolism, supporting that adipose tissue may play a crucial role in these adaptations, and thus ultimately in whole-body metabolic health and a reduction in cardiometabolic and metabolic problems [22,23]. In terms of inflammation, Ludzki and collaborators described that acute exercise, both moderate-intensity continuous exercise and high-intensity interval exercise, regulated the transcription of the inflammatory response of subcutaneous abdominal WAT in obese adults [24]. Acute exercise interventions have demonstrated that low- to moderate-intensity exercise can enhance whole-body fat oxidation, possibly by regulating adipose tissue lipolysis, gene expression of adipocytokines, or altering the cellular composition of adipose tissue [3]. However, there are limited data on the effect of chronic physical activity on inflammatory markers in obese adults, and the effectiveness of the response may depend on each individual’s characteristics [25]. On the other hand, there appears to be sex differences in fatty acid mobilisation during low and moderate intensity exercise, probably because of inherent physiological differences between the sexes [26]. Men and women have anatomical differences in the distribution of WAT and in the risk of developing obesity and its complications. Sex differences have been reported in WAT, including fat storage, adipocyte modelling and remodelling, lipolytic responses, and secondary inflammatory responses [27]. For example, sex chromosomes exert independent and interactive effects on adiposity, lipid metabolism, and lipo-inflammation [28]. Interestingly, Nigro et al. [29] have reported that chronic physical activity has a sexually dimorphic effect on subcutaneous WAT in mice, highlighting the importance of investigating both sexes.

While it is well known that the normal function of adipose tissue is disrupted in obesity, it remains unclear how exercise might regulate inflammatory markers and adipose tissue function, particularly gene expression, in obese adults. The aim is not only to focus on the changes in inflammatory gene expression induced by chronic exercise in WAT, but also to compare gene expression between women and men in response to exercise. Thus, we hypothesised that adapted program training for individuals with obesity enhances the expression of pro-inflammatory genes, irrespective of sex.

## 2. Materials and Methods

### 2.1. Participants

**Figure 1:**
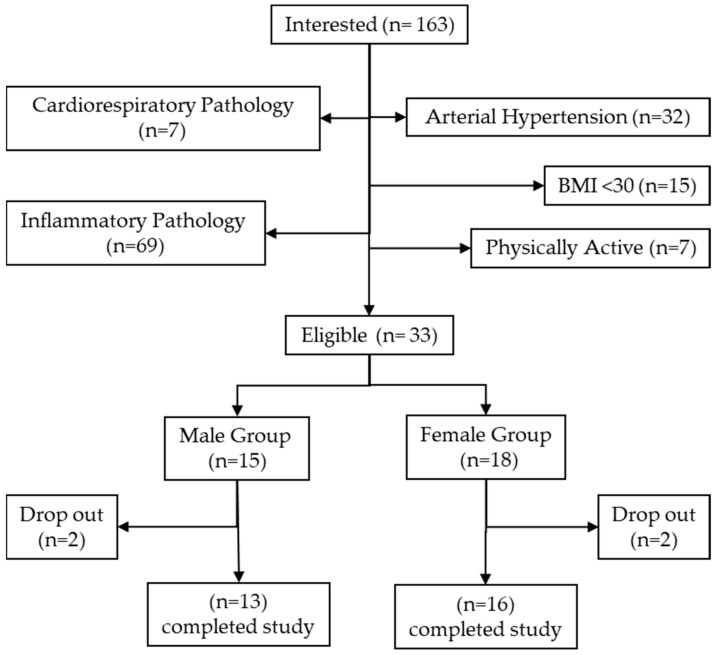
Recruitment flowchart.

This is a quasi-experimental, quantitative, longitudinal, pre–post intervention study. A total of 29 individuals participated in the study (13 men and 16 women), with an age range of 38 to 54 years (46.38 ± 4.66) and obesity (BMI = 36.05 ± 4.99 kg/m^2^). The following inclusion criteria were applied: (i) age 35–55 years; (ii) BMI: >30 kg/m^2^; (iii) abdominal circumference: >90 cm in women and >100 cm in men; (iv) fat percentage: >40 in women and >30 in men; (v) signed informed consent; and (vi) official medical authorisation allowing the participant to complete the planned exercise program. Due to heterogeneity, disparity in definitions, and the manifestation of obesity, it was established that participants meet at least two of criteria ii, iii, and iv. To determine body composition, the body composition analyser BIA InBody570^®^ (Bilbao, Spain) was used. Participants were excluded if (i) they had a diagnosis of pathology involving an inflammatory process (e.g., myocardial infarction in the last 12 months, rheumatoid arthritis, fibromyalgia, and osteoarthritis), and (ii) they did not complete 75% of the training sessions. All participants were informed about the objectives and procedures of the study and provided their written consent before enrolment. The study took place at the Research Centre on Physical and Sports Performance, Universidad Pablo de Olavide, Seville (Spain). The trial adhered to the guidelines of the Declaration of Helsinki (1989) of the World Medical Association, was approved by the bioethics committees of the Virgen Macarena and Virgen del Rocío University Hospitals (2544-N-21) and was registered in ClinicalTrials.gov (NCT05713461). Table 1 shows the baseline data and Figure 1 shows a flowchart of the selection process for participants in the study.

### 2.2. Study Design

**Figure 2:**
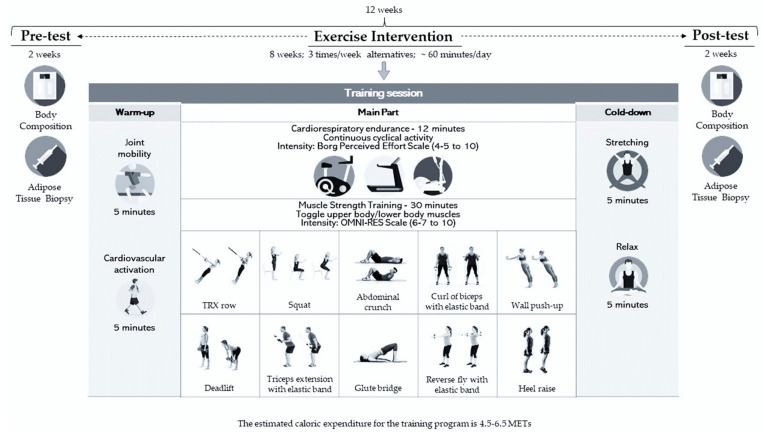
Summary of the training program and training session [30].

All participants followed a concurrent training program for 8 weeks, with three weekly sessions of ~60 min every other day between 5:30 pm and 8:30 pm (Figure 2).

### 2.3. Outcome Measures

Each training session consisted of 5 min of general joint mobility followed by 5 min of cardiovascular activation and 12 min of cardiorespiratory resistance exercise on a treadmill, cycle ergometer, or elliptical bike at moderate to low intensity. Intensity was determined using Borg’s rating of perceived exertion (RPE), which ranges from 4–5 to 10 [31]. Resistance training was then performed with 10 exercises of 45 s of activity and 45 s of rest during the first two weeks, and then 60 s and 30 s, respectively, from the third to the eighth week. Whereas in the first two weeks, two circuits with 2 min rests were completed in each session, after the third week, three circuits with 2 min rests were completed in each session. Each session ended with a cooling exercise. The intensity of the exercise was measured using the RPE OMNI-RES scale and was achieved and maintained between 6–7 and 10 [32]. A biopsy of subcutaneous abdominal adipose tissue was performed using the fine-needle aspiration technique at the beginning and the end of the intervention.

Total RNA from WAT samples was extracted using Trisure reagent (Bioline, Memphis, TN, USA) according to manufacturer’s instructions, and measured in a NanoDrop ND-1000 spectrophotometer (Thermo Scientific, Waltham, MA, USA). The A260/A280 ratio was used as a quality control. A two-step quantitative polymerase chain reaction (qPCR) protocol was carried out. Briefly, 1 µg of RNA was subjected to reverse transcription to obtain complementary DNA (cDNA) using iScript (Bio-Rad Laboratories), and 20 ng of cDNA was used as template for real-time PCR amplifications in a CFX96 system (Bio-Rad Laboratories, Hercules, CA, USA). In addition to cDNA, each PCR reaction contained the appropriate primer pairs for each candidate gene (Table 2) and brilliant SYBR green QPCR supermix (Bio-Rad Laboratories). Samples were run in triplicates. Quantification was performed using the standard delta 2−(ΔΔCt) method using the average of the threshold cycle (Ct) from triplicates of each gene. The 18S gene was used for normalisation within each sample. All graphs are presented as mean ± SEM using GraphPad software (GraphPad Software Inc., La Jolla, CA, USA).

### 2.4. Statistics

IBM SPSS Statistics 23 software (SPSS Inc., Chicago, IL, USA) was used for statistical analyses. For descriptive statistics, the mean and standard deviation were calculated. The reliability of the measurements was estimated with a 95% confidence interval for the mean. Two types of inferential analysis were performed: First, intra-group comparisons, involving pre-test vs. post-test analysis (men, women, and all). Because the samples did not meet the assumption of normality (Shapiro–Wilk), a Generalised Linear Model (GLM) was applied with the pre–post factor. Second, inter-group comparisons (men changes vs. women changes). Because the data did not meet the assumptions of normality (Shapiro–Wilk) and homoscedasticity (Levene), a Generalised Linear Model (GLM) was applied with the sex factor. Statistical significance was established for a value of *p* ≤ 0.05.

## 3. Results

**Figure 3:**
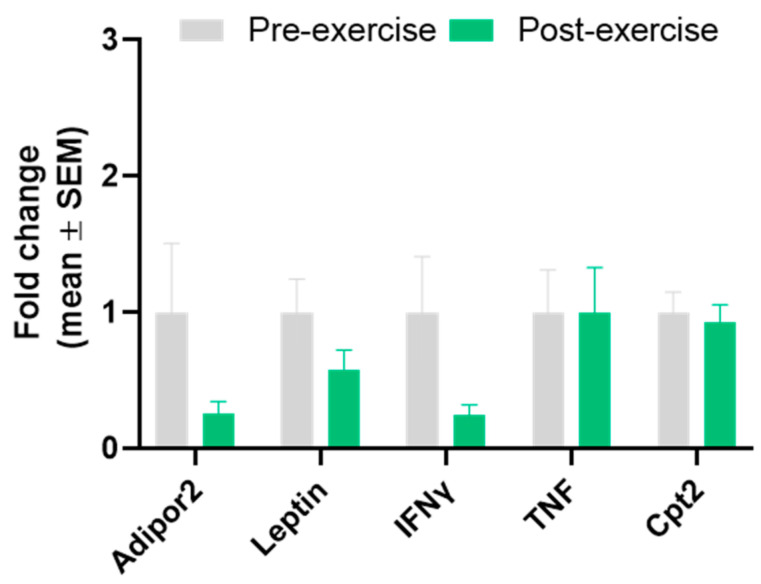
Gene expression of pro-inflammatory genes in subcutaneous WAT in men. Data are normalised with pre-intervention equal to 1. Results are presented as mean ± SEM.

Table 3 and Figure 3 show the normalised values and statistics before and after 8 weeks of exercise for pro-inflammatory genes of abdominal subcutaneous WAT from men. Although no significant differences were observed for ADIPOR2, leptin, TNFa, and CPT2, the expression of IFNg decreased slightly after exercise training.

**Figure 4:**
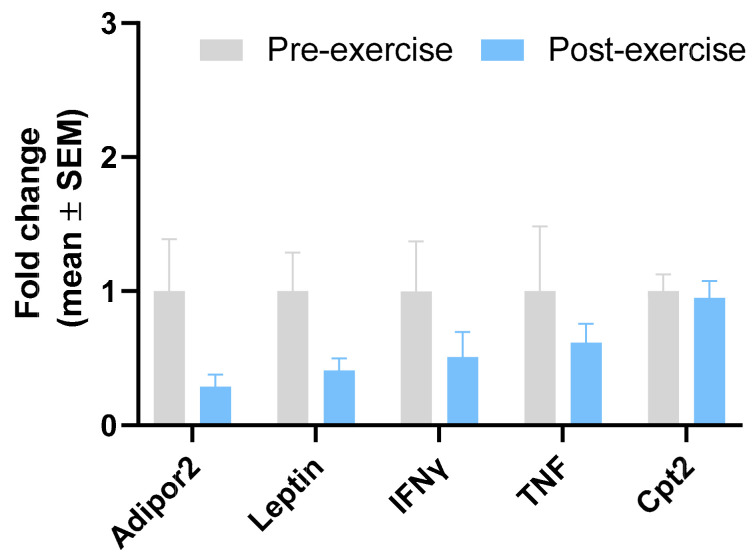
Gene expression of pro-inflammatory genes in subcutaneous WAT in women. Data are normalised with pre-intervention equal to 1. Results are presented as mean ± SEM.

Table 4 and Figure 4 show the normalised values and statistics before and after the intervention of the pro-inflammatory genes of the adipose tissue of the women. Leptin tended to decrease after 8 weeks of training (*p* = 0.057). No differences were observed for ADIPOR2, INFg, TNFa, and CPT2.

**Figure 5:**
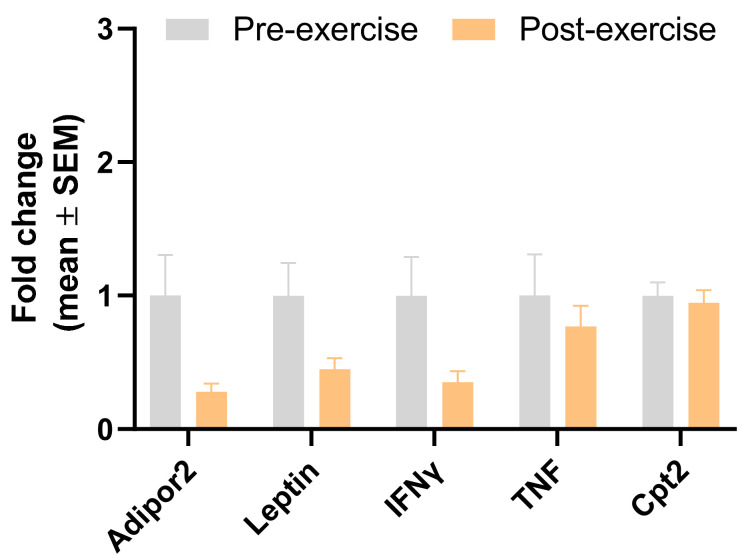
Gene expression of pro-inflammatory genes of subcutaneous WAT for all participants (n = 29). Data are normalised with pre-intervention equal to 1. Results are presented as mean ± SEM.

Table 5 and Figure 5 show the expression of genes analysed without considering sex (all participants). We can observe a significant downregulation of ADIPOR2 (*p* = 0.028), leptin (*p* = 0.041), and INFg (*p* = 0.040). No significant differences were found for TNFa and CPT2.

**Figure 6:**
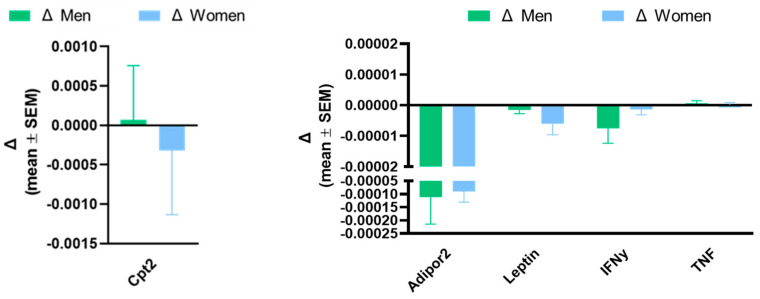
Comparison of changes between pre- and post-intervention in men and women for ADIPOR2, leptin, IFNg, TNFa, and CPT2 genes. Results are presented as mean ± SEM. No significant differences (GLM sex factor).

Figure 6 compares the pre–post changes in men with the pre–post changes in women. The results show there are no differences by sex in response to the physical training in the expression of the genes analysed (ADIPOR2: *p* = 0.823; Leptin: *p* = 0.245; IFNg: *p* = 0.181; TNFa: *p* = 0.738; CPT2: *p* = 0.713) using the Generalised Linear Model, pairwise.

## 4. Discussion

In this study, we aimed to determine whether chronic physical activity could attenuate subcutaneous WAT inflammation in obese individuals and, most importantly, to elucidate sex differences in response to exercise. The present study is a continuation of a previous investigation that analysed changes in body composition variables following the same training program. The results of that study showed significant improvements in all examined body composition variables. Specifically, fat mass, body weight, BMI, and vis-ceral fat area saw substantial reductions (*p* < 0.001), while fat-free mass and muscle mass experienced significant increases (*p* = 0.001) [33].

Table 5, which shows the raw data of the gene expression analysis, revealed a significant downregulation of ADIPOR2, leptin, and IFNg genes and a trend toward downregulation of TNFa and CPT2. Consistent with this, it has been reported that IFNg decreased in the blood of sedentary men after 24 weeks of exercise and in men diagnosed with type 2 diabetes who underwent 8 weeks of aerobic exercise [34,35]. In the same vein, Roh et al. (2020) [36] observed that 12 weeks of strength training reduced INFg in old, obese women. Taken together, these data highlight that INFg expression is downregulated by physical activity. Considering that IFNg is a pro-inflammatory factor, these results reinforce the hypothesis that physical exercise improves the ability to respond to chronic low-grade inflammation.

Most of the studies supporting the benefits of exercise in obesity are due to the downregulation of leptin and/or an improvement in leptin resistance [37]. Specifically, aerobic training for 3 months decreased plasma leptin levels without overall changes in plasma adiponectin levels and expression of adipose tissue genes [38]. In the same line, Bharath et al. (2018) reported weight loss, decreased waist circumference, and low levels of plasmatic leptin in obese individuals who underwent combined strength and aerobic training for 12 weeks [39]. Similarly, another study showed a negative association between leptin expression and exercise-induced weight loss [40]. However, each of these studies reported the effect of sex using the same training sessions. These results demonstrate that chronic physical activity negatively regulates the expression of the leptin gene, aligning with the findings of most published studies. Thus, it suggests that training yields benefits on regulatory factors associated with lipo-inflammation and insulin resistance. However, when the results were analysed in terms of sex, these differences were not statistically significant, likely due to the small group sizes.

Adiponectin (ADP) is one of the key adipokines with various beneficial effects related to obesity and metabolic syndromes, including improving glucose and lipid metabolism, enhancing insulin sensitivity, reducing oxidative stress and inflammation, promoting ceramides degradation, and stimulating adipose tissue vascularity [41]. In this line, adiponectin has been the subject of numerous studies analysing the effects of physical exercise and its role in obesity, insulin sensitivity, and low-grade inflammation. There is substantial evidence of the beneficial effects of exercise on adiponectin function in the context of obesity and chronic low-grade inflammation [42,43,44,45].

Many articles report an upregulation of the effect of exercise on adiponectin receptors and establish a positive association between ADIPOR2 (also ADIPOR1) and adiponectin. Blüher and co-workers observed an upregulation of adiponectin receptors after 4 weeks of training [46]. At the same time, O‘Leary described a 1.9-fold and 3.5-fold increase in muscular ADIPOR1 and ADIPOR2, respectively, after 12 weeks of training [47]. In adipose tissue, Blüher and co-workers also reported an upregulation of ADIPOR2 in the subcutaneous and visceral fat of obese and type 2 diabetic patients [48]. Interestingly, ADIPOR2 expression was correlated with adiponectin levels, lipid levels, insulin sensitivity, and glycaemic control [48]. In plasma, another study reported a two-fold higher expression of plasmatic ADIPOR1 after 12 weeks of high-intensity training [49].

However, the present study is the first to investigate ADIPOR2 gene expression between the sexes after physical activity. Likewise, the results show that ADIPOR2 was downregulated in both men and women after the intervention; however, these differences were not statistically significant in either sex (Table 3 and Table 4). When all participants were considered together, a significant difference was recorded post-intervention (Table 3 and Figure 6). This difference in statistical significance could be due to the small sample size. Consistent with our data, a recent study showed a trend toward decreased adiponectin and leptin expression in the subcutaneous fat of eight obese women [50]. Although evidence demonstrates that lower adiponectin levels and AdipoR1/R2 expression are associated with a higher incidence of T2D, in contrast, adults with T1D had significantly higher plasma adiponectin levels [51]. The downregulation of ADIPOR2 could be associated with decreased adiponectin activity and could, therefore, be interpreted as having a negative effect on insulin resistance or low-grade chronic inflammation However, the role of ADIPOR2 and its influence on adiponectin function is controversial, indicating that further research is necessary [52,53].

No studies investigate the response of CPT2 expression to physical training in human adipose tissue. CPT2 regulates the fatty acid oxidation and lipid metabolism that occurs mainly in the mitochondria of cells and is considered an anti-inflammatory factor [54]. Few papers have investigated its role in the exercise-induced benefits on obesity. A recent study discussed that taurine supplementation during exercise improves lipid metabolism in obese individuals [7]. Interestingly, another study observed an upregulation of muscle-derived CPT2 expression after 6 months of high/low intensity training [55]. In the same line, preclinical studies in Wistar rats showed an upregulation of CPT2 after 8 h of exercise or after high-intensity training when the rats were supplemented with L-carnitine [56]. In contrast, in our study, CPT2 expression was not altered in response to chronic exercise. CPT2 expression remained virtually unchanged after the intervention. Similarly, the results indicate that there are no differences between men and women. These findings are consistent with research conducted in rats undergoing aerobic exercise training [57]. Regulation of CPT2 by exercise probably requires more studies with longer intervention periods and different types of exercise programs.

It is well known that long-term exercise reduces pro-inflammatory cytokines such as TNF-alpha [58,59]. However, this effect seems to be regulated in a training-type-dependent manner, as high-intensity aerobic or resistance training for 10 weeks only showed a trend towards the downregulation of the regulatory TNFaIP3 gene [60]. In our study, adipose-derived TNFa expression was unchanged in response to chronic exercise. Similar results were obtained by Klimcakova et al. (2006) after strength training in obese individuals. In particular, the authors observed improved insulin sensitivity without changes in TNFa and leptin expression from abdominal visceral tissue [61]. On the other hand, TNFa and leptin, but not adiponectin, expression from subcutaneous fat showed a tendency to decrease after 12 weeks of moderate-to-low intensity training [38]. It should be pointed out that this study was conducted with eight participants, which could explain why the differences were not significant.

Significant differences in adipose tissue metabolism between women and men have been reported, making sex a variable that should always be considered when studying obesity and metabolic disorders [27]. However, the effect of sex in response to exercise remained unknown. Elucidating sex differences in genetics and epigenetics in response to exercise is crucial for designing personalised medicine using physical activity as a tool [62]. Indeed, Lebeck et al. reported sex differences in AQP7 expression (which is associated with a genetic predisposition to type 2 diabetes), in subcutaneous fat in response to exercise in women but not in men [63]. However, a recent study analysed the epigenetic pattern of MTHFR and showed no differences due to sex [64], confirming that further studies are needed to investigate the effect of sex. Regarding the regulation of the adipose inflammatory response by exercise, Da Mota et al. [65] reported a decrease in adiponectin and TNFa expression in both sexes, but only leptin was decreased in females after strength training. Therefore, one of the main points of this article is that we have investigated sex as a variable in the regulation of adipose tissue in obesity in response to exercise. Similar to Da Mota et al., we observed that leptin tends to be downregulated in women but not in men. However, most of the inflammatory genes analysed followed the same direction, suggesting that sex does not affect these genes differently. Related to this fact, we can find different studies about the effect of exercise on leptin in overweight and obese individuals. These studies often focus on single sexes. One meta-analysis in 2017 found only two studies including both men and women, but these had limitations [66]. Another meta-analysis in 2018 showed aerobic exercise increased adiponectin and decreased leptin levels in diabetic adults, but direct sex comparisons were lacking [67]. Moreover, recent research suggests exercise’s impact on inflammatory adipokines may not differ significantly between men and women [30].

Finally, we would like to mention that one of the limitations of this study is the absence of control groups. Due to the small sample size, we decided to prioritise two larger experimental groups to study the effect of sex on response to exercise, as there is a noted lack of information on this issue. Another limitation could be the duration of the intervention program; it is possible that the results would have been more pronounced with a longer period of physical training. Future research could examine the effects of exercise on subcutaneous white adipose tissue by comparing gene expression responses and plasma levels of lipo-inflammation indicators.

## 5. Conclusions

This study demonstrates that an adapted training program for individuals with obesity for 8 weeks downregulates the expression of pro-inflammatory genes in subcutaneous adipose tissue. Specifically, leptin and IFNg exhibited significant decreases. Regarding anti-inflammatory genes, CPT2 remained practically unchanged, while ADIPOR2 showed a significant decrease when both men and women were analysed together. However, when men and women were considered separately, no significant decreases were observed. Another notable finding is that the expression of genes analysed in response to exercise is independent of sex within the group of individuals with obesity studied.

## Figures and Tables

**Table 1:** Baseline data of the participants.

**ALL** (n = 29)						
	**Means**	**SD**	**CI (95%) LL**	**CI (95%) UL**	**Minimum**	**Maximum**
Age (year)	46.10	6.94	43.46	48.74	30.00	60.00
Hight (meter)	1.68	0.11	1.64	1.72	1.44	1.90
Weight (kg)	100.86	22.52	92.29	109.43	62.20	147.00
BMI (kg/m^2^)	35.47	5.44	33.40	37.54	28.02	49.69
BFM (kg)	43.95	13.18	38.93	48.96	24.00	79.30
PBF (%)	43.40	6.59	40.89	45.90	28.00	53.90
SMM (kg)	31.57	7.57	28.69	34.45	20.10	46.40
SMM (%)	31.50	3.92	30.01	32.99	25.25	40.65
FFM (kg)	56.61	12.83	51.73	61.49	37.40	82.30
FFM (%)	56.60	6.57	54.10	59.10	46.05	71.96
VFA (cm^2^)	204.82	49.14	186.13	223.51	106.80	316.40
Systolic PA (mmHg)	133.10	17.15	126.57	139,62	94.00	163.00
Diastolic PA (mmHg)	84.31	12.67	79.49	89.13	59.00	119.00
Glycaemia (mg/dL)	104.59	24.73	95.18	113.99	55.00	175.00
**FEMALE** (n = 13)						
	**Mean**	**SD**	**CI (95%) LL**	**CI (95%) UL**	**Minimum**	**Maximum**
Age (year)	45.81	7.78	41.67	49.96	30.00	60.00
Hight (meter)	1.61	0.08	1.57	1.66	1.44	1.74
Weight (kg)	93.19	23.42	80.71	105.67	62.20	147.00
BMI (kg/m^2^)	35.46	6.30	32.10	38.81	28.02	49.69
BFM (kg)	44.43	14.84	36.52	52.34	24.80	79.30
PBF (%)	47.11	4.73	44.58	49.63	39.00	53.90
SMM (kg)	26.66	5.42	23.77	29.55	20.10	38.30
SMM (%)	29.09	2.53	27.75	30.44	25.25	33.29
FFM (kg)	48.38	9.25	43.45	53.30	37.40	67.70
FFM (%)	52.91	4.74	50.39	55.43	46.05	61.03
VFA (cm^2^)	208.71	50.47	181.81	235.60	129.90	316.40
Systolic PA (mmHg)	125.25	16.25	116.59	133.90	94.00	158.00
Diastolic PA (mmHg)	79.81	14.44	72.11	87.50	59.00	119.00
Glycaemia (mg/dL)	105.44	21.62	93.91	116.96	67.00	159.00
**MALE** (n = 16)						
	**Mean**	**SD**	**CI (95%) LL**	**CI (95%) UL**	**Minimum**	**Maximum**
Age (year)	46.46	6.04	42.81	50.11	38.00	56.00
Hight (meter)	1.76	0.08	1.71	1.81	1.60	1.90
Weight (kg)	110.31	17.97	99.45	121.16	85.60	140.00
BMI (kg/m^2^)	35.49	4.42	32.82	38.16	28.27	42.30
BFM (kg)	43.35	11.37	36.48	50.23	24.00	58.40
PBF (%)	38.83	5.69	35.39	42.27	28.00	48.80
SMM (kg)	37.62	5.01	34.59	40.64	28.60	46.40
SMM (%)	34.46	3.27	32.48	36.43	28.71	40.65
FFM (kg)	66.74	8.71	61.48	72.00	51.00	82.30
FFM (%)	61.14	5.66	57.72	64.56	51.20	71.96
VFA (cm^2^)	200.04	49.05	170.40	229.68	106.80	253.70
Systolic PA (mmHg)	142.76	17.71	134.81	150.72	116.00	163.00
Diastolic PA (mmHg)	89.85	7.33	85.41	94.28	82.00	105.00
Glycaemia (mg/dL)	103.54	28.98	86.03	121.05	55.00	175.00

BMI (Body Mass Index); BFM (Body Fat Mass); PBF (Percent Body Fat); SMM (Skeletal Muscle Mass); FFM (Fat Free Mass); VFA (Visceral Fat Area).

**Table 2:** Sequences of RT-qPCR primers for gene expression analysis.

Target	Expressed Molecule Role	GenBank Accession	Direction	Sequence (5′→3′)
**ADIPOR2**	Adiponectin Receptor Anti-inflammatory	NM_024551	Forward Reverse	ACCAAGGAGATTTGGAGCCC GGACATGCCCATAAAGCCCT
**CPT2**	Beta-oxidation of long-chain fatty acids in mitochondria. Anti-inflammatory	NM_000098	Forward Reverse	CATACAAGCTACATTTCGGGACC AGCCCGGAGTGTCTTCAGAA
**IFNg**	Immune regulation Pro-inflammatory	NM_000619	Forward Reverse	TCGGTAACTGACTTGAATGTCCA TCGCTTCCCTGTTTTAGCTGC
**Leptin**	Anorexigenic Pro-inflammatory	NM_000230	Forward Reverse	AAACGCAAAGGGCTGAAAGC AGATCGCAGTCACCAGTGTG
**TNFa**	Regulation of multiple mechanisms of the immune response. Pro-inflammatory	NM_021833	Forward Reverse	CAATCACCGCTGTGGTAAAAAC GTAGAGGCCGATCCTGAGAGA
**RPS18s**	house-keeping	NM_022551	Forward Reverse	CGATGGGCGGCGGAAAATA TTGGTGAGGTCAATGTCTGCT

ADIPOR2: Adiponectin receptor-2; CPT2: Carnitine palmitoyltransferase-2; IFNg: Interferon-gamma; TNFa: Tumour necrosis factor-alpha; RPS18s: Ribosomal protein S18.

**Table 3:** Normalised scores and statistics before/after the interventions for men (n = 13).

	Pre-Exercise		Post-Exercise		
	Mean ± SD	CI (95%)	Mean ± SD	IC (95%)	*p*-Value ^§^
**ADIPOR2**	1 ± 1.825	(−0.103–2.103)	0.260 ± 0.284	(0.069–0.451)	0.16
**Leptin**	1 ± 0.874	(0.472–1.528)	0.577 ± 0.482	(0.253–0.900)	0.135
**IFNg**	1 ± 1.476	(0.108–1.892)	0.250 ± 0.241	(0.088–0.411)	0.083
**TNFa**	1 ± 1.116	(0.326–1.674)	0.922 ± 1.112	(0.245–1.739)	0.986
**CPT2**	1 ± 0.537	(0.676–1.324)	0.924 ± 0.443	(0.626–1.221)	0.695

^§^ *p*-value for GLM pre–post factor. ADIPOR2: Adiponectin receptor-2; IFNg: Interferon-gamma; TNFa: Tumour necrosis factor-alpha; CPT2: Carnitine palmitoyltransferase-2.

**Table 4:** Normalised scores and statistics before/after the interventions for women (n = 19).

	Pre-Exercise		Post-Exercise		
	Mean ± SD	CI (95%)	Mean ± SD	IC (95%)	*p*-Value ^§^
**ADIPOR2**	1 ± 1.504	(0.167–1.833)	0.291 ± 0.320	(0.097–0.484)	0.084
**Leptin**	1 ± 1.120	(0.380–1.620)	0.408 ± 0.321	(0.214–0.602)	0.057
**INFg**	1 ± 1.443	(0.201–1.799)	0.509 ± 0.674	(0.102–0.916)	0.244
**TNFa**	1 ± 1.875	(−0.039–2.039)	0.616 ± 0.504	(0.311–0.920)	0.458
**CPT2**	1 ± 0.493	(0.727–1.273)	0.939 ± 0.484	(0.646–1.231)	0.773

^§^ *p*-value GLM pre–post factor. ADIPOR2: Adiponectin receptor-2; IFNg: Interferon-gamma; TNFa: Tumour necrosis factor-alpha; CPT2: Carnitine palmitoyltransferase-2.

**Table 5:** Normalised scores and statistics before/after the interventions for all participants (n = 29).

	Pre-Exercise		Post-Exercise		
	Mean ± SD	CI (95%)	Mean ± SD	IC (95%)	*p*-Value ^§^
**ADIPOR2**	1 ± 1.609	(0.376–1.624)	0.279 ± 0.308	(0.149–0.409)	0.028
**Leptin**	1 ± 1.295	(0.498–1.502)	0.448 ± 0.401	(0.279–0.618)	0.041
**INFg**	1 ± 1.529	(0.407–1.593)	0.351 ± 0.411	(0.177–0.524)	0.040
**TNFa**	1 ± 1.633	(0.367–1.633)	0.768 ± 0.766	(0.445–1.092)	0.516
**CPT2**	1 ± 0.517	(0.799–1.201)	0.946 ± 0.473	(0.750–1.141)	0.685

^§^ *p*-value for GLM pre–post factor. ADIPOR2: Adiponectin receptor-2; IFNg: Interferon-gamma; TNFa: Tumour necrosis factor alpha; CPT2: Carnitine palmitoyltransferase-2.

## Data Availability

Data in the research data repository of the University of Seville—awaiting DOI assignment.
